# Weight development between age 5 and 10 years and its associations with dietary patterns at age 5 in the ABCD cohort

**DOI:** 10.1186/s12889-020-08559-y

**Published:** 2020-04-01

**Authors:** Viyan Rashid, Martinette T. Streppel, Marielle F. Engberink, Peter J. M. Weijs, Mary Nicolaou, Arnoud P. Verhoeff

**Affiliations:** 1 Department of Nutrition and Dietetics, Faculty of Sports and Nutrition, Amsterdam University of Applied Sciences, Dr. Meurerlaan 8, Amsterdam, SM 1067 The Netherlands; 2 Department Nutrition & Dietetics, Internal Medicine, Amsterdam University Medical Centers, location VUmc ZH4A12. De Boelelaan 1117, Amsterdam, HV 1081 The Netherlands; 3 Department of Public Health, Amsterdam UMC, University of Amsterdam, Amsterdam Public Health Research Institute, Meibergdreef 9, Amsterdam, AZ 1105 The Netherlands; 4 Department of Sociology, University of Amsterdam, Nieuwe Achtergracht 166, Amsterdam, WV 1018 The Netherlands; 5 Department of Epidemiology, Health Promotion and Health Care Innovation, Public Health Service Amsterdam, Nieuwe Achtergracht 100, Amsterdam, WT 1018 The Netherlands

**Keywords:** BMI, Body mass index, Thinness, Normal weight, Overweight, Obesity, Socio-economic position, Ethnicity, Children, Young children

## Abstract

**Background:**

Social inequalities in bodyweight start early in life and track into adulthood. Dietary patterns are an important determinant of weight development in children, towards both overweight and underweight. Therefore, we aimed to examine weight development between age 5 and 10 years by ethnicity, SES and thereafter by BMI category at age 5, to explore its association with dietary patterns at age 5.

**Methods:**

Participants were 1765 children from the Amsterdam Born Children and their Development (ABCD) cohort that had valid data on BMI at age 5 and 10 and diet at age 5. Linear mixed model analysis was used to examine weight development between age 5 and 10 years and to assess if four previously identified dietary patterns at age 5 (snacking, full-fat, meat and healthy) were associated with weight development. Analyses were adjusted for relevant confounders, stratified by ethnicity and SES and thereafter stratified per BMI category at age 5.

**Results:**

Overall, weight decreased in Dutch and high SES children and increased in non-Dutch and low/middle SES children. Across the range of bodyweight categories at age 5, we observed a conversion to normal weight, which was stronger in Dutch and high SES children but less pronounced in non-Dutch and low/middle SES children. Overall, the observed associations between weight development and dietary patterns were mixed with some unexpected findings: a healthy dietary pattern was positively associated with weight development in most groups, regardless of ethnicity and SES (e.g. Dutch B 0.084, 95% CI 0.038;0.130 and high SES B 0.096, 95% CI 0.047;0.143) whereas the full-fat pattern was negatively associated with weight development (e.g. Dutch B -0.069, 95% CI -0.114;-0.024 and high SES B -0.072, 95% CI -0.119;-0.026).

**Conclusions:**

We observed differential weight development per ethnic and SES group. Our results indicate that each ethnic and SES group follows its own path of weight development. Associations between dietary patterns and weight development showed some unexpected findings; follow-up research is needed to understand the association between dietary patterns and weight development.

## Background

In recent years, research and Public Health has focussed on the high prevalence of obesity and overweight among children, mainly present in ethnic minority and lower Socio-economic (SES) groups [1–4]. Concurrently, there is evidence of a growing prevalence of underweight in children of native origin in high-income countries [5, 6]. Although it is not clear how important this trend is for clinical practice, both underweight and overweight/obesity are a risk factor for health problems in later life [7, 8]. Therefore it’s important to focus on the total range of bodyweight.

Social inequality in bodyweight starts early in life and tracks into adulthood. Children who are already overweight or obese in mid-childhood are likely to remain so by the start of adolescence [9, 10], and later into adulthood [11] and associations between rapid infancy growth and obesity in childhood were strongest in populations consisting of greater proportions of ethnic minority and low SES children [12]. In the Netherlands ethnic differences in overweight are present from 2 to 4 years onwards [1, 2, 13, 14]. Data from the Amsterdam Born Children and their Development (ABCD) cohort observed ethnic differences in overweight at age 2 [1] and overweight at this age was, specifically in non-Dutch children, a strong predictor of staying overweight (tracking) at age 5–6 years [1, 15]. In a Brittish birth cohort study, differences in BMI trajectories were found between 4 year old children from different maternal education background. And these differences widened with increasing age [16].

Studying the total range of weight development in high-income countries is a relatively new focus in Public Health research and studies are lacking. Based on the observation that underweight is increasing in prevalence in Europe [5, 6], it is important to also consider how this tracks in childhood and whether it is asociated with ethnicity and/or SES. Earlier results in the ABCD cohort showed that both ethnicity and SES were related to different dietary patterns at age 5 [17].

There is growing interest in the role of dietary patterns in early childhood as a determinant of obesity risk [18]. Diets of poor quality might be important, as they are often characterized by high consumption of energy-dense foods [19–21]. Children with higher intakes of food from unhealthy patterns showed an increase in total and central body adiposity [22]. Remarkably, in 9–10 year old Norwegian children a higher prevalence of overweight and obesity was found in children consuming dietary patterns identified as healthy, and less overweight was found in children adhering to a dietary pattern high in processed foods [23, 24]. Therefore, it is of interest to confirm the association between dietary patterns and weight develoment in the ABCD cohort. The aims of this study were to examine (1) weight development between age 5 and 10 years by ethnicity, SES and thereafter by BMI category at age 5 and (2) its association with dietary patterns at age 5.

## Methods

### Study design and population

**Fig. 1:**
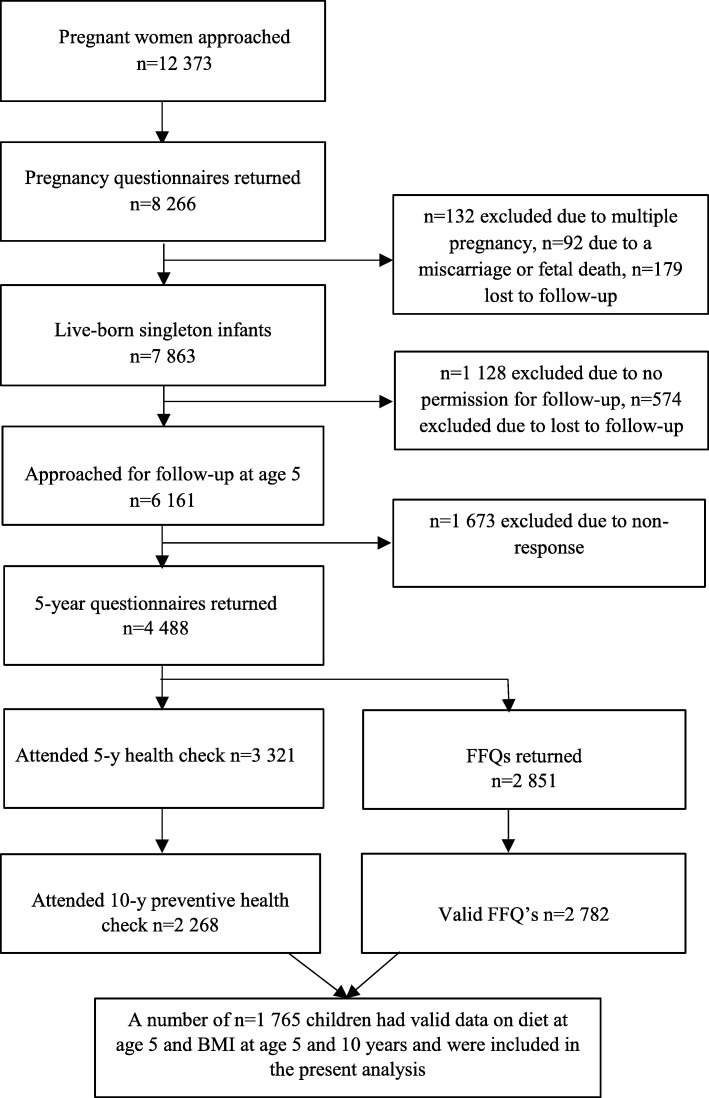
Flow-chart of the sampling procedure of the ABCD study population (*n* = 1765)

Data were used from the ABCD cohort, a large ongoing community-based birth cohort (http://www.abcd-study.nl/). The cohort study design has been described previously [25]. In brief, between January 2003 and March 2004, all pregnant women living in Amsterdam, the Netherlands, were invited to participate in the ABCD cohort by their obstetric care provider at their first parental care visit. Of the 12,373 women approached, 8266 women filled out a pregnancy questionnaire that covered socio-demographic characteristics, obstetric history and lifestyle characteristics. The questionnaire was available in Dutch, English, Turkish and the Arabic language. When the children turned 5 years of age, the addresses of 6161 mothers were retrieved from the Youth Health Care registry. A 5-year questionnaire was sent to the woman’s home address and 4488 questionnaires were filled out by the mothers. These women received a invitation for a health check and a self-administered Food Frequency Questionnaire (FFQ) for their children. The health check was completed by 3321 children and a number of 2851 mothers returned the FFQ. At age 10, parents received an invitation for a regular preventive health check of their child, provided by the department of Youth Health Care of the Public Health Service Amsterdam carried out at their primary school. Finaly, a number of 1765 children had valid data on diet at age 5 and BMI at age 5 and 10 years, and were included in the present analysis (Fig. 1).

### Assessment of BMI

At age 5, height and weight were measured during the ABCD health check were children were measured by a team of trained researchers according to standard protocols using a Leicester portable height measure (Seca) and a Marsden weightening scale (model MS-4102), respectively. At age 10, height and weight were measured by a health professional during the regular preventive health check at the children’s primary school. During both measurements, children were dressed in light clothing.

Data on height and weight were converted to Body Mass Index (BMI) scores [weight (kg) / height (m)^2^]. BMI scores were classified in age- and sex-specific BMI cut-offs from the International Obesity Task Force [7, 26] and three categories were formed: underweight, normal weight and overweight/obesity. BMI scores were also converted to age- and sex-specific BMI z-scores (SD scores), by comparison with the ‘World Health Organization standards’ [27].

### Assessment of dietary patterns

The assessment of dietary patterns within the ABCD cohort at age 5 has been previously described [24]. Mothers filled in a validated 71-item FFQ developed by TNO Food (Zeist, The Netherlands) [28]. In brief, per food item, consumption frequency, portion size and the type of product consumed over the last 4 weeks was reported. Frequency options were “never”, “less than once a week”, “once a week”, “2-3 times a week”, “4-5 times a week”, and “6-7 times a week”. Mothers were given the possibility to fill in food items that were not included in the FFQ. Based on the data clearance protocol developed by TNO Food, the returned FFQs were scanned and the data on amounts (g/day) of products consumed and intake of energy was calculated using the Dutch Food Composition Database (NEVO) 2010 [29]. Energy adjusted intake (g/d) of 41 composed food groups were derived to assess dietary patterns, using Principal Component Analyses (PCA) with varimax rotation. Four dietary patterns were derived: a snacking (sweet and savory snacks, refined breakfast products and low intakes of whole-grain breakfast products), full-fat (full-fat spreads and pasta dishes and low intakes of low-fat spreads), meat (low and high fat meat, sauces and refined grain products for warm meals) and healthy pattern (water and tea, vegetables, fish and fruits). Individuals were given a pattern score for each dietary pattern as a sum of the 41 standardized food group intakes, each weighted according to their factor loading. Positive pattern scores indicate higher consumption of food groups in that pattern.

### Assessment of ethnicity and socio-economic status

Data on ethnicity was collected via the pregnancy questionnaire and based on the country of birth of the pregnant woman and her mother including both first-generation women (born outside the Netherlands) and second generation women (born in the Netherlands but whose mother was born in another country). Five ethnic categories were formed: Dutch, African Surinamese, Turkish, Moroccan and “other” ethnicities (mainly of non-western origin). Level of maternal education was collected via the 5-year questionnaire and was used as a proxy for SES. It was defined as the highest education completed: low: (a few years of) primary education / lower secondary education / lower vocational education, middle: general higher secondary education / intermediate vocational education or high: higher professional programmes or university programmes leading to a bachelor or master degree [30].

### Assessment of potential confounders

Covariates were selected on the basis of theory and previous literature and were included in case of a significant change (≥10%) effect estimated on the dietary patterns. Potential covariates that might influence weight development and its relation with dietary patterns were sex, children’s exact age at the 5-years health check, screen time at age 5 and maternal BMI at age 5. Maternal BMI (kg/m2) was based on mothers self-reported height and weight when the child was 5-year old and screen time (hour/day) was based on the duration in hours that the child spent watching TV or used a computer or console per day at age 5 [31].

### Statistical analysis

Population and antropometric characteristics were described in numbers, percentages or means with standard deviations (SD) for the total population and by ethnic and SES groups separately. Of the children that were included in this analyses (*n* = 1765), 1765 children had complete data on sex, age and ethnicity, 1759 on SES, 1756 on screen time and 1666 on maternal BMI.

For the first research objective, linear mixed model analyses were used to examine the change in BMI z-scores over time (from age 5 to 10 years) (i.e. weight development) in the total population by including time as a fixed effect. This method incorporates all available repeated measurements of the BMI z-scores simultaneously and takes into account that these measurements are correlated within participants. The likelihood ratio tests were used to determine a suitable random effect structure. The model with a random intercept and slope with time was considered most appropriate for all analyses. We tested for interactions between weight development between age 5 and 10 years and ethnic and SES groups by including an interaction term of ethnic and SES groups with time (significant at a *p*-value< 0.10). No significant interactions were found between the low and middle SES groups, therefore we reported the results for these groups combined. We further examined weight development between age 5 and 10 years by ethnicity and SES. (Model 1). Adjustments were made for children’s sex, exact age, screen time, and maternal BMI, ethnicity or SES (Model 2). Model 2 was further stratified by BMI categories at age 5: underweight (Model 2a), normal weight (Model 2b) and overweight/obesity (Model 2c). In sensitivity analysis, we tested for interaction between weight development between age 5 and 10 years and sex within the total population, by including an interaction term of sex with time.

For the second research objective, linear mixed effect models were used to examine the association between dietary patterns at age 5 and BMI z-scores at age 5 and 10 years (i.e.weight development) by ethnicity and SES (Model 1). Adjustments were made for children’s sex, age, screen time, and maternal BMI, ethnicity or SES (Model 2). We performed two sensitivity analyses. First, Model 2 was additionally adjusted for total energy intake, in order to obtain insight into the role of energy intake in this relationship. Second, per ethnic and SES group, Model 2 was further stratified by BMI categories at age 5: underweight (Model 2a), normal weight (Model 2b) and overweight/obesity (Model 2c). All statistical analyses were performed in SPSS version 24 for windows and the level of statistical significance was set at 0.05.

## Results

### Population characteristics

Population characteristics of the total study population (*n* = 1765) including stratification by ethnicity and SES groups are presented in Table 1. Mean (SD) age was 5.7 (0.5) y at the 5-years health check and 10.6 (0.4) y at the 10-years health check. Most children were from Dutch origin (79.3%), followed by Moroccan (5.4%), African Surinamese (4.4%), Turkish (2.6%) and “other” ethnicities (8.3%). The majority of children (68.3%) belonged to the high SES group and 31.7% belonged to the low/middle SES group. Highest BMI z-scores at age 5 were found in Turkish (0.60 SD 0.99) and Moroccan children (0.55 SD 1.67) and lowest BMI z-scores were found in Dutch (− 0.04 SD 0.86) and high SES children (− 0.06 SD 0.81).

**Table 1:**
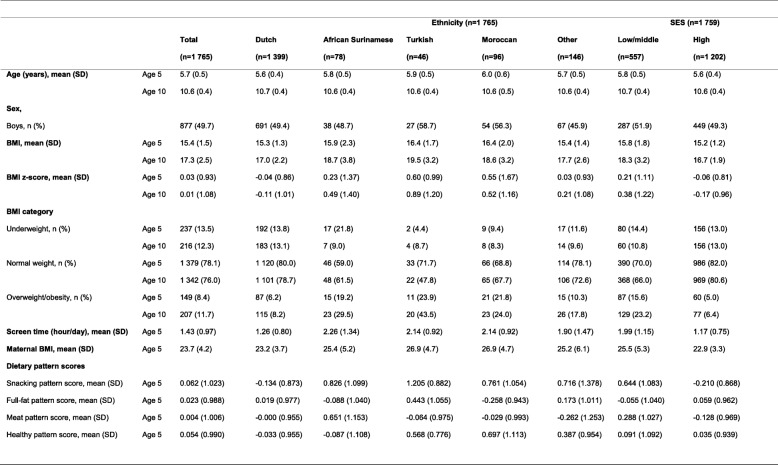
Population characteristics at age 5 and 10 years by ethnicity and SES in the ABCD study population (*n* = 1765)

**Fig. 2:**
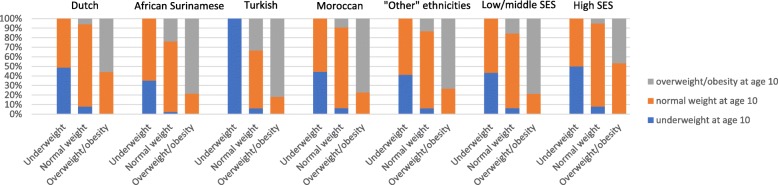
Change over BMI categories between age 5 and 10 years by ethnicity and SES in the ABCD study population (*n* = 1765)

Results in Fig. 2 presents the change over BMI categories between age 5 and 10 years. In most groups, the majority of children that were underweight at age 5 switched to normal weight at age 10 (e.g. 64% in African Surinamse and 56% in Moroccan children). However, in Dutch and high SES children, respectively 51 and 50% of the children switched to normal weight and a higher percetage of children remained underweight at age 10. In children that were normal weight at age 5, mainly African Surinamese (24%) and Turkish children (33%) switched to the overweight/obesity category at age 10 (e.g. 9% in Moroccan children and 16% in low/middle SES children). In none of the groups, children switched from underweight to overweight/obesity or from overweight/obesity to underweight.

Mean (SD) BMI z-scores per BMI category at age 5 and 10 years are presented in Additional file 1. In 10-year old overweight/obese children, mean (SD) BMI z-scores were lower in Dutch (1.44 SD 0.71) and high SES children (1.25 SD 0.61) than in other groups (e.g. Moroccan: 1.85 SD 0.63 and low/middle SES: 1.89 SD 0.66). Antropometric characteristics further specified by sex are shown in Additional file 2.

### Weight development between age 5 and 10 years

Within the total population (*n* = 1765), there was a non-significant decrease in weight between age 5 and 10 years (B -0.018, 95% CI -0.052; 0.016). When this model was adjusted for confounders, the association was comparable (B -0.021, 95% CI -0.056; 0.014).

### Weight development by ethnicity

Significant interactions were found between weight development and most ethnic groups; between Dutch and African Surinamese, Dutch and Turkish, Dutch and children of “other” ethnicities (*p* < 0.001), between Moroccan and African Surinamese (*p* = 0.022), Moroccan and Turkish (*p* = 0.011) and between high and low/middle SES children (p < 0.001).

**Fig. 3:**
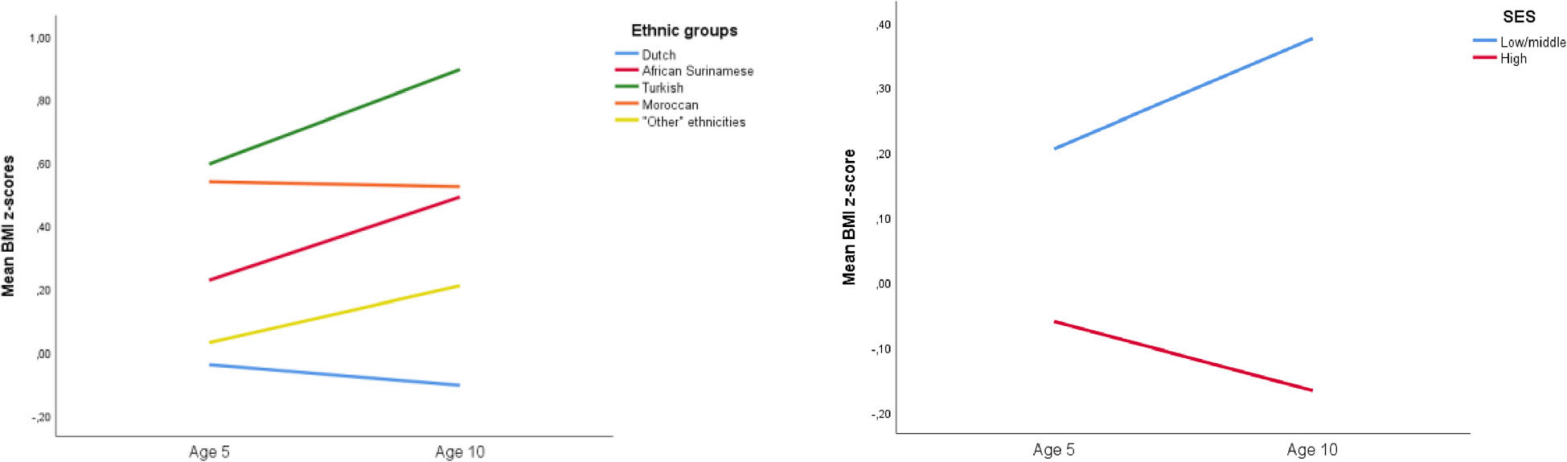
Observed mean BMI z-scores at age 5 and 10 years by ethnicity and SES in the ABCD study population (*n* = 1765)

Therefore analyses of weight development between age 5 and 10 years are shown per ethnic group seperately (Table 2 and Fig. 3). In Dutch children, weight decreased (B -0.070, 95% CI -0.107; − 0.033; Model 2). In Moroccan children there was no change in weight (B -0.001, 95% CI -0.182; 0.179). In African Surinamese, Turkish and children of “other” ethnicities weight increased. Weight developement differed depending on children’s BMI category at age 5. In children that were underweight at age 5 we observed an increase in weight across all ethnic groups (e.g. in Dutch children: B 0.186, 95% CI 0.092; 0.280; Model 2a). In children with a normal weight at age 5 we observed a decrease in weight in Dutch and Moroccan children, although in the latter group this was not statistically significant. In Turkish, African Surinamese and children of “other” ethnicities however we observed an increase in weight (e.g. B 0.455, 95% CI 0.175; 0.734 in Turkish children). In overweight/obese children we observed a decrease in weight except in the Turkish or “other” ethnicities groups.

**Table 2:**
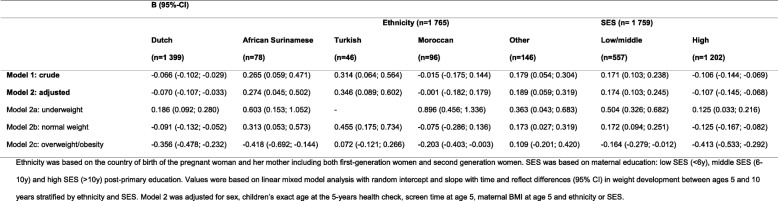
Weight development between age 5 and 10 years by ethnicity and SES in the ABCD study population (*n* = 1765)

### Weight development by SES

Analyses of weight development between age 5 and 10 years per SES group are presented in Table 2 and Fig. 3. In low/middle SES children, weight increased (B 0.174, 95% CI 0.103; 0.245; Model 2) while in high SES children weight decreased (B -0.107, 95% CI -0.145; − 0.068). In children that were underweight at age 5 we observed an increase in weight in both SES groups. In children with a normal weight at age 5 we observed an increase in weight in low/middle SES children and a decrease in high SES children. In overweight/obese children we observed a decrease in weight in both SES groups.

Sensitivity analyses showed that weight development was significant different between boys (B 0.056, 95% CI 0.005; 0.106) and girls (B -0.097, − 0.145; 95% CI − 0.049) in the total population (*p* < 0.001).

### Dietary patterns and weight development by ethnicity

Associations between dietary patterns at age 5 and weight development between age 5 and 10 years per ethnic group are presented in Table 3. In Dutch children, full-fat pattern scores at age 5 were negatively associated with weight development between age 5 and 10 years (B -0.069, 95% CI -0.114; − 0.024; Model 2), while healthy pattern scores were positively associated with weight development (B 0.092, 95% CI 0.047; 0.137). Also in Moroccan children, healthy pattern scores were positively associated with weight development (B 0.259, 95% CI 0.002; 0.516).

**Table 3:**
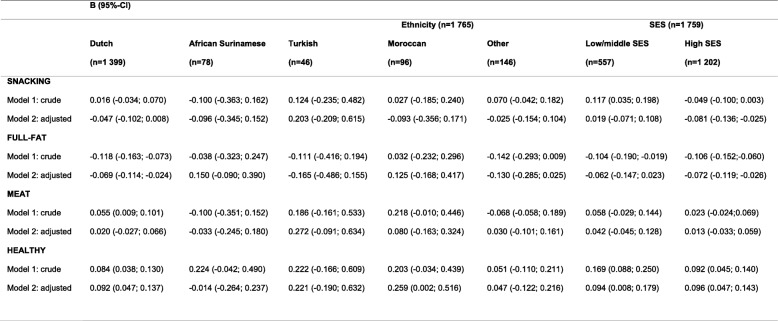
The association between dietary patterns at age 5 and weight development between age 5 and 10 years by ethnicity and SES (*n* = 1765)

Other results were not statistically significant but showed comparable associations: also in Turkish and children of “other” ethnicities, we observed that full-fat pattern scores were negatively associated, and healthy pattern scores positively associated with weight development. In most ethnic groups, snacking pattern scores were negatively associated and meat pattern scores were positively associated with weight development between age 5 and 10 years.

### Dietary patterns and weight development by SES

The associations between dietary patterns at age 5 and weight development between age 5 and 10 years by SES group are presented in Table 3. In low/middle SES children, healthy pattern scores at age 5 were positively associated with weight development (B 0.094, 95% CI 0.008; 0.179; Model 2). In high SES children, snacking (B -0.081, 95% CI -0.136; − 0.025) and full-fat pattern scores (B -0.072, 95% CI -0.119; − 0.026) were negatively associated with weight development while healthy pattern scores were positively associated with weight development (B 0.096, 95% CI 0.047; 0.143).

### Sensitivity analyses dietary patterns and weight development by BMI category

Dietary patterns were associated with weight development, independently of energy intake: additional adjustment for total energy intake (B reflects the intake per kcal) did not change the results (e.g. in Dutch children, full-fat pattern scores were B -0.069, 95% CI -0.116; − 0.026 without adjustment for total energy intake and B -0.071, 95% CI -0.116; − 0.026 with adjustment for total energy intake). In the same model, energy intake was not associated with weight development in ethnic or SES groups (e.g. B -0.0003, 95% CI -0.0010; 0.0004 in Turkish children and B -0.0001, 95% CI -0.0005; 0.0002 in children of “other” ethnicities). Finally, when Model 2 was further stratified by BMI categories at age 5: underweight (Model 2a), normal weight (Model 2b) and overweight/obesity (Model 2c), no clear pattern was found among children in different BMI categories. In normal weight Dutch children, healthy pattern scores were associated with positive weight development (B 0.070, 95% CI 0.035; 0.106). And in normal weight high SES children, snacking pattern scores were negatively associated with weight development (B -0.051, 95% CI -0.096; − 0.006) and healthy pattern scores were associated with positive weight development (B 0.070, 95% CI 0.031; 0.109) between age 5 and 10 years. Other results were mainly non-significant associations without a clear structure.

## Discussion

This study revealed differences in weight development between age 5 and 10 years between ethnic and SES groups in the large multi-ethnic ABCD cohort, consisting of 1765 children, indicating that these groups follow different patterns of weight development. In Dutch and high SES children we observed a decreased weight development, while in non-Dutch and low/middle SES children weight development increased. Across the range of bodyweight categories at age 5, we observed a conversion to normal weight, which was stronger in Dutch and high SES children but less pronounced in non-Dutch and low/middle SES children. Dutch and high SES children were more often underweight or normal weight, at both ages and less often overweight/obese compared to non-Dutch and low/middle SES children. Contrary to our expectations, high healthy pattern scores were postitively associated with weight development in Dutch, Moroccan and all SES categories of children and high full-fat pattern scores were negatively associated with weight development in Dutch and high SES children.

In line with our results, other studies also showed ethnic [8, 32] and SES [24, 33] differences in overweight and obesity. Prevalence of overweight/obesity was nearly double in children of non-western origin compared to children of western origin [8, 32]. However in our study, we also observed clear differences in the prevalence of overweight/obesity within the non-native group. At age 10, 44% of the children from Turkish origin was overweight/obese while 30% of children of African Surinamese and 24% of Moroccan origin were overweight/obese. Ethnicity also carries information about SES. Maternal educational level (in our study used as a proxy for SES) explains part of the ethnic differences in childhood overweight [14]. Non-native groups are more often from lower SES groups than native groups. In our study, 23% of Dutch children was from the low/middle SES group, compared to 69% in African Surinamese, 87% in Turkish, 82% in Moroccan children and 43% in children of “other” ethnicities. Despite comparable SES levels, we clearly observed that Turkish children developed more often overweight/obesity between the age of 5 and 10 years (44%) than Moroccan children (24%). These results suggest that the non-native group is a diverse group with possibly different causes and mechanisms that contribute to childhood overweight/obesity in each ethnic group.

We observed a higher tendency to underweight and normal weight in Dutch and high SES children. Another Dutch study also observed higher percentages of underweight in Dutch children, while percentages of underweight in children of Turkish and Moroccan origin were at most only half as high [34]. But unlike our results, this study did not observe SES differences in underweight rates [34]. A study in 4–5 year old children in Norway, found comparable percentages of underweight between children of European (10.4%) and Middle East/North African origin (12.7%) [32]. These differences could be possibly due to unintended side effects of current obesity prevention programs, and current delivery of these programs to lower SES groups is either not adequately reaching into these groups [35].

Aditionally we examined weight development per ethnic and SES groep, stratified by BMI category at age 5. We observed a conversion to normal weight in most groups, which is not in line with a study in 6–12 year old Norwegian children, where they found that overweight children tended to gain more weight than normal-weight children [24]. But the conversion to normal weight differed per ethnic and SES group. It was stronger in Dutch and high SES children that were more likely to experience negative weight development, regardless of BMI category at age 5. Observing weight development stratified per BMI category at age 5, showed that none of the children switched from underweight to overweight/obesity or from overweight/obesity to thinnes. We also obtained more specific information of the differences in weight development between the non-native groups. Weight development of Moroccan children, seemed to be more in line with the structure of weight development in Dutch and high SES children. In normal weight Dutch and high SES children, weight development was negative while in normal weight African Surinamese, Turkish, “other” ethnicities and low/middle SES children, weight development was positive. In normal weight Moroccan children, weight decreased; however results were not statistically significant. In overweight/obese children, we observed a further increase of weight in Turkish children and children of “other” ethnicities.

Our results indicate that a healthy dietary pattern at age 5 is postively associated with weight development whereas a full-fat dietary pattern at age 5 is negatively associated weight development. Oellingrath et al. [23] also observed a positive association between dietary patterns identified as healthy (a ‘varied Norwegian’ pattern characterized by intakes of fish and meat for dinner, brown bread, regular white or brown cheese, lean meat, fish spread, and fruit and vegetable) and BMI. In this study, 9 year old children with high scores on this ‘varied Norwegian’ pattern were more likely to be overweight or obese at 9 years of age [23]. Another Dutch birth cohort observed that better diet quality at age 1 and 8 years was associated with higher height, weight, and FFMI, but not with body fatness up to age 10 years. This was independent of diet quality at an earlier or later time point [36]. We did not find other studies in the literature that observed an association between a dietary pattern comparable to our full-fat pattern (high intakes of full-fat cheese and full-fat spreads, low intakes of low-fat cheese, low-fat spreads and low-fat dairy) and BMI. In line with other studies [23, 37–40], we did not observe a clear association between the snacking pattern (a dietary pattern characterized by snacking items) and weight development. Overall, the observed patterns of association between dietary patterns and weight development were mixed, also after analyses were stratified by BMI category at age 5. We observed only a few statistically significant associations, mainly within the larger groups (e.g. Dutch, low/middle SES and high SES). Therefore based on these results, we cannot draw any conslusions about the role of dietary patterns at the age of 5 in weight development over the next 5 years.

Childhood obesity is a complex construct. A diversity of factors contribute as biological- and lifestyle factors but also the social- and obesogenic environment [41]. There is also interest in the role of dietary patterns in early childhood as a determinant of obesity risk [18]. Few studies observed associations between dietary patterns and overweight [22–24]. In our analyses we considered sex, children’s exact age at the 5-years health check, screen time at age 5 and maternal BMI at age 5 as major determinants that might influence the association between dietary patterns at age 5 and weight development.

Additionally, we studied the role of energy intake in the association between dietary patterns at age 5 and weight development. A previous study by Northstone et al. [42] also failed to observe differences in outcome when adjusting for energy intake before entry into the PCA analysis. However, to exclude a possible role of energy intake, we included energy adjusted intake of food groups as an input in the PCA analyses to derive dietary patterns [43]. In sensitivity analyses, we also observed that additionally adjusting for energy intake in the association between dietary patterns at age 5 and weight development did not change the results. In the same Model, we observed that energy intake was not associated with weight development in the ethnic and SES groups.

Results of the association between dietary patterns and weight development stratified by ethnic and SES group, also give information about the possible moderating effect of ethnicity and SES. In another sensitivity analysis, we studied the intermediate effect of dietary patterns on the association with ethnicity / SES on weight development and we observed that the intermediate role of dietary patterns on weight development is limited. However, in our cohort, results were more pronounced in the ethnic groups than in the SES groups.

### Methodological considerations

A strength of this study is the population based cohort-design that included a number of 1765 children with complete data on dietary patterns at age 5 and BMI at age 5 and 10 years, of different ethnic and SES groups that are often excluded in epidemiological studies. Analyses stratified per BMI category gave an complete overview of weight development in these groups. Height and weight were measured by a team of trained researchers and health professionals according to standard protocols.

However, interpretation of the association between dietary patterns and weight development is limited due to the cross sectional nature of dietary intake assessment. Dietary tracking, the maintenance of a dietary pattern over a certain time period, has been observed during childhood and from childhood to adolescence [40]. Another limitation of our study is that the FFQ was based on food commonly consumed by the Dutch population as determined by the Food Consumption Survey 1997–1998 [44]. The FFQ was validated with the gold standard of doubly labelled water in a group of 4 to 6-year-old children, although this validation study did not include non-Dutch groups [45]. Thus the FFQ may not reflect the food intake of ethnic minority children. To account for this we included an open question at the end of the FFQ, asking participants to note commonly eaten foods that were missing from the questionnaire. However, in the analysis we decided to not include the food items mentioned in the open item section because it was used only by a few mothers and it was unclear whether the mentioned items were additional to what was already included in the FFQ. In sub-analysis we found that energy intake related to energy requirements (based on Schofield resting metabolism) was not different between Dutch and non-Dutch groups, implying that the FFQ is able to capture total food intake in this population. Smaller numbers in ethnic groups is inherent to the ABCD cohort design but it is possible that some biases may have been introduced into the analyses, particularly as the non-responders tended to come disproportionately from lower SES and ethnic minority groups. Response rates per ethnic and SES group were 27% for Dutch, 12% for African Surinamese, 10% for Turkish, 13% for Moroccan, 13% for “other” ethnicities, 11% for low/middle SES and 38% for high SES. A nonresponse analysis determining the degree of selective response and selection bias between pregnancy and birth outcomes, indicated that selective non-response was present in the ABCD cohort, but selection bias was acceptably low and did not influence the studied birth outcomes [46].

## Conclusions

We observed ethnic and SES differences in weight development between age 5 and 10 years in a multi-ethnic, population-based cohort of 5–10 year old children in The Netherlands. Our results indicate that each ethnic and SES group follows its own path of weight development. Results of the role of dietary patterns in weight development were mixed, limiting our ability to draw conclusions regarding the exact role of dietary patterns in ethnic and SES differences in weight development. Overall, however, our results indicate that a healthy pattern is positively associated, and a full-fat pattern is negatively associated with weight development.

## Supplementary information

**Additional file 1. **Mean (SD) BMI z-scores per BMI category at age 5 and 10 years by ethnicity and SES in the ABCD study population (*n* = 1765).

**Additional file 2. **Antropometric characteristics at age 5 and 10 years by ethnicity, SES and sex in the ABCD study population (*n* = 1765).

## Data Availability

The datasets generated and/or analysed during the current study are not publicly available due ethical restrictions related to protecting patient confidentially but are available from the corresponding author on reasonable request.

## Abbreviations

BMI: Body Mass Index
ABCD cohort: Amsterdam Born Children and their Development cohort
SES: Socio-economic status
FFQ: Food Frequency Questionnaire
PCA: Principal Component Analysis
SD: Standard deviations
e.g: For example

## References

[1] de Hoog ML, van Eijsden M, Stronks K, Gemke RJ, Vrijkotte TG. Overweight at age two years in a multi-ethnic cohort (ABCD study): the role of prenatal factors, birth outcomes and postnatal factors. BMC Public Health. 2011. 10.1186/1471-2458-11-611. 10.1186/1471-2458-11-611 PMC3171368 21806791

[2] de Wilde JA, van Dommelen P, Middelkoop BJ, Verkerk PH (2009). Trends in overweight and obesity prevalence in Dutch, Turkish, Moroccan and Surinamese south Asian children in the Netherlands. Arch Dis Child.

[3] Outcome Monitor Amsterdamse Aanpak gezond Gewicht. Staat van gezond gewicht en leefstijl van Amsterdamse kinderen. Amsterdam Municipality (2017). https://jongerenopgezondgewicht.nl/userfiles/Meten/outcome_monitor_aanpak_gezond_gewicht_2017.pdf. Accessed 2 Apr 2019.

[4] van Dommelen P, Schönbeck Y, HiraSing RA, van Buuren S (2015). Call for early prevention: prevalence rates of overweight among Turkish and Moroccan children in the Netherlands. Eur J Pub Health.

[5] Franssen SJ, van der Wal MF, Jansen P, van Eijsden M (2015). Thinness and overweight in children from Amsterdam: a trend analysis and forecast. Ned Tijdschr Geneeskd.

[6] Garrido-Miguel M, Cavero-Redondo I, Álvarez-Bueno C, Rodriguez-Artalejo F, Moreno Aznar L, Ruiz JR, et al. Prevalence and trends of thinness, overweight and obesity among children and adolescents aged 3-18 years across Europe: a protocol for a systematic review and meta-analysis. BMJ Open. 2017. 10.1136/bmjopen-2017-018241. 10.1136/bmjopen-2017-018241 PMC5778300 29273660

[7] Cole TJ, Flegal KM, Nicholls D, Jackson AA (2007). BMJ.

[8] Wake M, Clifford SA, Patton GC, Waters E, Williams J, Canterford L, Carlin JB (2013). Morbidity patterns among the underweight, overweight and obese between 2 and 18 years: population-based cross-sectional analyses. Int J Obes.

[9] Reilly JJ, Bonataki M, Leary SD, Wells JC, Davey-Smith G, Emmett P, et al. Progression from childhood overweight to adolescent obesity in a large contemporary cohort. Int J Pediatr Obes. 2011. 10.3109/17477166.2010.497538. 10.3109/17477166.2010.497538 20883104

[10] Wright CM, Emmett PM, Ness AR, Reilly JJ, Sherriff A. Tracking of obesity and body fatness through mid-childhood. Arch Dis Child. 2010. 10.1136/adc.2009.164491. 10.1136/adc.2009.164491 20522467

[11] Reilly JJ, Kelly J (2011). Long-term impact of overweight and obesity in childhood and adolescence on morbidity and premature mortality in adulthood: systematic review. Int J Obes.

[12] Andrea SB, Hooker ER, Messer LC, Tandy T, Boone-Heinonen J. Does the association between early life growth and later obesity differ by race/ethnicity or socioeconomic status? A systematic review. Ann Epidemiol. 2017. 10.1016/j.annepidem.2017.08.019. 10.1016/j.annepidem.2017.08.019 PMC6688753 28911983

[13] Fredriks AM, Van Buuren S, Sing RA, Wit JM, Verloove-Vanhorick SP (2005). Alarming prevalences of overweight and obesity for children of Turkish, Moroccan and Dutch origin in the Netherlands according to international standards. Acta Paediatr.

[14] van Rossem L, Hafkamp-de Groen E, Jaddoe VW, Hofman A, Mackenbach JP, Raat H. The role of early life factors in the development of ethnic differences in growth and overweight in preschool children: a prospective birth cohort. BMC Public Health. 2014. 10.1186/1471-2458-14-722. 10.1186/1471-2458-14-722 PMC4227130 25022314

[15] de Hoog ML, van Eijsden M, Stronks K, Gemke RJ, Vrijkotte TG. Ethnic differences in cardiometabolic risk profile at age 5-6 years: the ABCD study. PLoS One. 2012. 10.1371/journal.pone.0043667. 10.1371/journal.pone.0043667 PMC3423381 22916294

[16] Howe LD, Tilling K, Galobardes B, Smith GD, Ness AR, Lawlor DA. Socioeconomic disparities in trajectories of adiposity across childhood. Int J Pediatr Obes. 2011. 10.3109/17477166.2010.500387. 10.3109/17477166.2010.500387 PMC5102325 20860432

[17] Rashid V, Engberink MF, van Eijsden M, Nicolaou M, Dekker LH, Verhoeff AP, et al. Ethnicity and socioeconomic status are related to dietary patterns at age 5 in the Amsterdam born children and their development (ABCD) cohort. BMC Public Health. 2018. 10.1186/s12889-017-5014-0. 10.1186/s12889-017-5014-0 PMC5759294 29310648

[18] Smithers LG, Golley RK, Brazionis L, Lynch JW. Characterizing whole diets of young children from developed countries and the association between diet and health: a systematic review. Nutr Rev. 2011. 10.1111/j.1753-4887.2011.00407.x. 10.1111/j.1753-4887.2011.00407.x 21790612

[19] Fisk CM, Crozier SR, Inskip HM, Godfrey KM, Cooper C, Robinson SM (2011). Southampton Women's survey study group. Influences on the quality of young children's diets: the importance of maternal food choices. Br J Nutr.

[20] Ambrosini GL, Emmett PM, Northstone K, Howe LD, Tilling K, Jebb SA (2012). Identification of a dietary pattern prospectively associated with increased adiposity during childhood and adolescence. Int J Obes.

[21] Rose CM, Birch LL, Savage JS. Dietary patterns in infancy are associated with child diet and weight outcomes at 6 years. Int J Obes. 2017. 10.1038/ijo.2017.27. 10.1038/ijo.2017.27 28133360

[22] Vieira-Ribeiro SA, Andreoli CS, Fonseca PCA, Miranda Hermsdorff HH, Pereira PF, Ribeiro AQ, et al. Dietary patterns and body adiposity in children in Brazil: a cross-sectional study. Public Health. 2019. 10.1016/j.puhe.2018.10.002. 10.1016/j.puhe.2018.10.002 30500570

[23] Oellingrath IM, Svendsen MV, Brantsaeter AL. Eating patterns and overweight in 9- to 10-year-old children in Telemark County, Norway: a cross-sectional study. Eur J Clin Nutr. 2010. 10.1038/ejcn.2010.152. 10.1038/ejcn.2010.152 PMC3002052 20717128

[24] Skår A, Meza TJ, Fredriksen PM. Development of weight and height in Norwegian children: the health oriented pedagogical project (HOPP). Scand J Public Health. 2018. 10.1177/1403494818769852. 10.1177/1403494818769852 29754577

[25] van Eijsden M, Vrijkotte TG, Gemke RJ, van der Wal MF (2011). Cohort profile: the Amsterdam born children and their development (ABCD) study. Int J Epidemiol.

[26] Cole TJ, Lobstein T. Extended international (IOTF) body mass index cut-offs for thinness, overweight and obesity. Pediatr Obes. 2012. 10.1111/j.2047-6310.2012.00064.x. 10.1111/j.2047-6310.2012.00064.x 22715120

[27] WHO Multicentre Growth Reference Study Group (2006). WHO child growth standards: length/height-for-age, weight-for-age, weight-for-length, weight-for-height and body mass index-for-age: methods and development.

[28] Dutman AE, Stafleu A, Kruizinga A, Brants HA, Westerterp KR, Kistemaker C, et al. Validation of an FFQ and options for data processing using the doubly labelled water method in children. Public Health Nutr. 2011. 10.1017/S1368980010002119. 10.1017/S1368980010002119 20707949

[29] RIVM (2010). National Institute for public health and the environment.

[30] CBS (2016). Standaard onderwijsindeling 2016/2017.

[31] Chinapaw MJ, Altenburg TM, van Eijsden M, Gemke RJ, Vrijkotte TG (2014). Screen time and cardiometabolic function in Dutch 5-6 year olds: cross-sectional analysis of the ABCD-study. BMC Public Health.

[32] Toftemo I, Jenum AK, Lagerløv P, Júlίusson PB, Falk RS, Sletner L. Contrasting patterns of overweight and thinness among preschool children of different ethnic groups in Norway, and relations with maternal and early life factors. BMC Public Health. 2018. 10.1186/s12889-018-5952-1. 10.1186/s12889-018-5952-1 PMC6108110 30139343

[33] Barriuso L, Miqueleiz E, Albaladejo R. Socioeconomic position and childhood-adolescent weight status in rich countries: A systematic review, 1990–2013. BMC Pediatr. 2015. 10.1186/s12887-015-0443-3. 10.1186/s12887-015-0443-3 PMC4578240 26391227

[34] Schönbeck Y, van Dommelen P, HiraSing RA, van Buuren S (2015). Thinness in the era of obesity: trends in children and adolescents in the Netherlands since 1980. Eur J Pub Health.

[35] Hardy LL, Mihrshahi S, Gale J, Drayton BA, Bauman A, Mitchell J (2017). 30-year trends in overweight, obesity and waist-to-height ratio by socioeconomic status in Australian children, 1985 to 2015. Int J Obes.

[36] Nguyen AN, Jen V, Jaddoe VWV, Rivadeneira F, Jansen PW, Ikram MA, et al. Diet quality in early and mid-childhood in relation to trajectories of growth and body composition. Clin Nutr. 2019. 10.1016/j.clnu.2019.03.017. 10.1016/j.clnu.2019.03.017 30967308

[37] Shi Z, Makrides M, Zhou SJ. Dietary patterns and obesity in preschool children in Australia: a cross-sectional study. Asia Pac J Clin Nutr. 2018. 10.6133/apjcn.032017.19. 10.6133/apjcn.032017.19 29384330

[38] Durão C, Severo M, Oliveira A, Moreira P, Guerra A, Barros H, et al. Association between dietary patterns and adiposity from 4 to 7 years of age. Public Health Nutr. 2017. 10.1017/S1368980017000854. 10.1017/S1368980017000854 PMC10261466 28534458

[39] Kiefte-de Jong JC, de Vries JH, Bleeker SE, Jaddoe VW, Hofman A, Raat H (2013). Socio-demographic and lifestyle determinants of ‘Western-like’and ‘health conscious’dietary patterns in toddlers. Br J Nutr.

[40] Oellingrath IM, Svendsen MV, Brantsaeter AL. Tracking of eating patterns and overweight - a follow-up study of Norwegian schoolchildren from middle childhood to early adolescence. Nutr J. 2011. 10.1186/1475-2891-10-106. 10.1186/1475-2891-10-106 PMC3200168 21978299

[41] Gurnani M, Birken C, Hamilton J (2015). Childhood obesity: causes, consequences, and management. Pediatr Clin N Am.

[42] Northstone K, Ness AR, Emmett PM, Rogers IS (2008). Adjusting for energy intake in dietary pattern investigations using principal components analysis. Eur J Clin Nutr.

[43] Willett WC, Howe GR, Kushi LH (1997). Adjustment for total energy intake in epidemiologic studies. Am J Clin Nutr.

[44] Anon. Zo eet Nederland, 1998. Food consumption survey 1997–1998. Den Haag: Voedingscentrum 1998.

[45] Dutman AE, Stssafleu A, Kruizinga A, Brants HA, Westerterp KR, Kistemaker C (2011). Validation of an FFQ and options for data processing using the doubly labelled water method in children. Public Health Nutr.

[46] Tromp M, van Eijsden M, Ravelli AC, Bonsel GJ (2009). Anonymous non-response analysis in the ABCD cohort study enabled by probabilistic record linkage. Paediatr Perinat Epidemiol.

