# Supplementary material for: Weight development between age 5 and 10 years and its associations with dietary patterns at age 5 in the ABCD cohort
Source: BMC Public Health. 2020 Apr 1;20:427. doi: 10.1186/s12889-020-08559-y (PMC7110614; doi:10.1186/s12889-020-08559-y)
Supplement: Supplementary file 1 — Additional file 1. Mean (SD) BMI z-scores per BMI category at age 5 and 10 years by ethnicity and SES in the ABCD study population (n = 1765). [file 12889_2020_8559_MOESM1_ESM.pdf]

Additional file 1. Mean (SD) BMI z-scores per BMI category at age 5 and 10 years by ethnicity and SES in the ABCD study population (n=1 765).

		Ethnicity (n=1 765)					SES (n= 1 759)	
		**Dutch (n=1 399)**	**African Surinamese (n=78)**	**Turkish (n=46)**	**Moroccan (n=96)**	**Other (n=146)**	**Low/middle (n=557)**	**High (n=1 202)**
**BMI category**								
Underweight, n (%)	**Age 5**	-1.36 (0.39)	-1.44 (0.46)	-1.74 (0.69)	-1.57 (0.42)	-1.47 (0.36)	-1.44 (0.05)	-1.35 (0.34)
	**Age 10**	-1.20 (0.82)	-0.88 (1.09)	-1.47 (0.33)	-0.82 (0.91)	-1.04 (0.90)	-0.99 (1.00)	-1.25 (0.74)
Normal weight, n (%)	**Age 5**	0.05 (0.57)	0.18 (0.70)	0.29 (0.49)	0.29 (0.55)	0.04 (0.60)	0.14 (0.58)	0.04 (0.57)
	**Age 10**	-0.04 (0.85)	0.51 (1.15)	0.67 (1.06)	0.26 (0.92)	0.19 (0.88)	0.32 (0.96)	0.08 (0.83)
Overweight/obesity, n (%)	**Age 5**	1.77 (0.49)	2.27 (0.72)	1.91 (0.57)	2.14 (0.63)	1.68 (0.47)	2.02 (0.59)	1.64 (0.37)
	**Age 10**	1.44 (0.71)	1.98 (0.72)	1.98 (0.57)	1.85 (0.63)	1.75 (0.76)	1.89 (0.66)	1.25 (0.61)

Ethnicity was based on the country of birth of the pregnant woman and her mother including both first-generation women and second generation women. SES was based on maternal education: low SES (<6y), middle SES (6-10y) and high SES (>10y) post-primary education. BMI categories were based on classification of Cole [7, 26] (n=1 765).
