# Supplementary material for: Weight development between age 5 and 10 years and its associations with dietary patterns at age 5 in the ABCD cohort
Source: BMC Public Health. 2020 Apr 1;20:427. doi: 10.1186/s12889-020-08559-y (PMC7110614; doi:10.1186/s12889-020-08559-y)
Supplement: Supplementary file 2 — Additional file 2. Antropometric characteristics at age 5 and 10 years by ethnicity, SES and sex in the ABCD study population (n = 1765). [file 12889_2020_8559_MOESM2_ESM.pdf]

Additional file 2. Antropometric characteristics at age 5 and 10 years by ethnicity, SES and sex in the ABCD study population (n=1 765).

| | | Ethnicity (n=1 765) | | | | | SES (n= 1 759) | |
|---|---|---|---|---|---|---|---|---|
| | | **Dutch (n=1 399)** | **African Surinamese (n=78)** | **Turkish (n=46)** | **Moroccan (n=96)** | **Other (n=146)** | **Low/middle (n=557)** | **High (n=1 202)** |
| **BMI, mean (SD)** | **Age 5** | **15.3 (1.3)** | **15.9 (2.3)** | **16.4 (1.7)** | **16.4 (2.0)** | **15.4 (1.4)** | **15.8 (1.8)** | **15.2 (1.2)** |
| | Boys | 15.3 (1.2) | 16.2 (2.0) | 16.2 (1.4) | 16.3 (2.0) | 15.5 (1.5) | 15.7 (1.7) | 15.3 (1.1) |
| | Girls | 15.3 (1.4) | 15.5 (2.6) | 16.6 (2.1) | 16.5 (2.2) | 15.4 (1.3) | 15.8 (2.0) | 15.2 (1.3) |
| | **Age 10** | **17.0 (2.2)** | **18.7 (3.8)** | **19.5 (3.2)** | **18.6 (3.2)** | **17.7 (2.6)** | **18.3 (3.2)** | **16.7 (1.9)** |
| | Boys | 17.0 (2.0) | 19.2 (3.6) | 19.6 (3.2) | 18.4 (3.1) | 17.7 (2.7) | 18.1 (2.9) | 16.9 (1.9) |
| | Girls | 17.0 (2.4) | 18.2 (4.1) | 19.3 (3.2) | 18.8 (3.3) | 17.7 (2.5) | 18.5 (3.4) | 16.7 (2.0) |
| **BMI z-score, mean (SD)** | **Age 5** | **-0.04 (0.86)** | **0.23 (1.37)** | **0.60 (0.99)** | **0.55 (1.67)** | **0.03 (0.93)** | **0.21 (1.11)** | **-0.06 (0.81)** |
| | Boys | -0.06 (0.87) | 0.53 (1.31) | 0.56 (0.89) | 0.54 (1.22) | 0.05 (1.02) | 0.20 (1.13) | -0.05 (0.83) |
| | Girls | -0.02 (0.86) | -0.06 (1.36) | 0.65 (1.16) | 0.54 (1.11) | 0.01 (0.86) | 0.21 (1.09) | -0.07 (0.79) |
| | **Age 10** | **-0.11 (1.01)** | **0.49 (1.40)** | **0.89 (1.20)** | **0.52 (1.16)** | **0.21 (1.08)** | **0.38 (1.22)** | **-0.17 (0.96)** |
| | Boys | -0.04 (1.01) | 0.79 (1.35) | 1.00 (1.25) | 0.53 (1.19) | 0.27 (1.15) | 0.39 (1.24) | -0.06 (0.97) |
| | Girls | -0.17 (1.01) | 0.20 (1.40) | 0.75 (1.16) | 0.52 (1.15) | 0.15 (1.02) | 0.36 (1.20) | -0.27 (0.93) |
| **BMI category** | | | | | | | | |
| Underweight, n (%) | **Age 5** | **192 (13.8)** | **17 (21.8)** | **2 (4.4)** | **9 (9.4)** | **17 (11.6)** | **81 (14.5)** | **156 (13.0)** |
| | Boys | 99 (14.3) | 6 (15.8) | 1 (3.7) | 5 (9.3) | 10 (14.9) | 44 (15.2) | 77 (13.1) |
| | Girls | 94 (13.3) | 11 (27.5) | 1 (5.3) | 4 (9.5) | 7 (8.9) | 37 (13.8) | 79 (12.8) |
| | **Age 10** | **183 (13.1)** | **7 (9.0)** | **4 (8.7)** | **8 (8.3)** | **14 (9.6)** | **60 (10.8)** | **156 (13.0)** |
| | Boys | 70 (10.1) | 2 (5.3) | 3 (11.1) | 5 (9.3) | 6 (9.0) | 29 (10.1) | 57 (9.8) |
| | Girls | 113 (16.0) | 5 (12.5) | 1 (5.3) | 3 (7.2) | 8 (10.1) | 31 (11.6) | 99 (16.1) |
| Normal weight, n (%) | **Age 5** | **1 120 (80.0)** | **46 (59)** | **33 (71.7)** | **66 (68.8)** | **114 (78.1)** | **338 (69.7)** | **986 (82.0)** |
| | Boys | 561 (81.2) | 24 (63.2) | 22 (81.5) | 38 (7.4) | 51 (76.1) | 205 (70.9) | 489 (83.4) |
| | Girls | 558 (78.8) | 22 (55.0) | 11 (57.9) | 27 (64.3) | 63 (79.7) | 183 (68.3) | 497 (80.7) |

| | | | | | | | | |
|---|---|---|---|---|---|---|---|---|
| | **Age 10** | **1 101 (78.7)** | **48 (61.5)** | **22 (47.8)** | **65 (67.7)** | **106 (72.6)** | **368 (66.0)** | **969 (80.6)** |
| | Boys | 567 (82.1) | 24 (63.2) | 12 (44.4) | 36 (66.7) | 49 (73.1) | 197 (68.2) | 489 (83.4) |
| | Girls | 534 (75.4) | 24 (60.0) | 10 (52.6) | 29 (69.0) | 57 (72.2) | 171 (63.8) | 480 (77.9) |
| Overweight/obesity, n (%) | **Age 5** | **87 (6.2)** | **15 (19.2)** | **11 (23.9)** | **21 (21.8)** | **15 (10.3)** | **88 (15.8)** | **60 (5.0)** |
| | Boys | 31 (4.5) | 8 (21.1) | 4 (14.8) | 11 (20.4) | 6 (9.0) | 40 (13.8) | 20 (3.4) |
| | Girls | 56 (7.9) | 7 (17.5) | 7 (36.8) | 11 (26.2) | 9 (11.4) | 48 (17.9) | 40 (6.5) |
| | **Age 10** | **115 (8.2)** | **23 (29.5)** | **20 (43.5)** | **23 (24.0)** | **26 (17.8)** | **129 (23.2)** | **77 (6.4)** |
| | Boys | 54 (7.8) | 12 (31.6) | 12 (7.4) | 13 (24.1) | 12 (17.9) | 63 (21.8) | 40 (6.5) |
| | Girls | 61 (8.6) | 11 (27.5) | 8 (42.1) | 10 (23.8) | 14 (17.7) | 66 (24.6) | 37 (6.0) |

Ethnicity was based on the country of birth of the pregnant woman and her mother including both first-generation women and second generation women. SES was based on maternal education: low SES (<6y), middle SES (6-10y) and high SES (>10y) post-primary education. BMI categories were based on classification of Cole [7, 26] (n=1 765).
